# Delineation of the *Pasteurellaceae*-specific GbpA-family of glutathione-binding proteins

**DOI:** 10.1186/1471-2091-12-59

**Published:** 2011-11-16

**Authors:** Bjorn Vergauwen, Ruben Van der Meeren, Ann Dansercoer, Savvas N Savvides

**Affiliations:** 1 Unit for Structural Biology, Laboratory for Protein Biochemistry and Biomolecular Engineering (L-ProBE), Department of Biochemistry and Microbiology, Ghent University, 9000 Ghent, Belgium

**Keywords:** glutathione, GbpA, HbpA, DppA, solute-binding protein, SBP, ABC transporter

## Abstract

**Background:**

The Gram-negative bacterium *Haemophilus influenzae *is a glutathione auxotroph and acquires the redox-active tripeptide by import. The dedicated glutathione transporter belongs to the ATP-binding cassette (ABC)-transporter superfamily and displays more than 60% overall sequence identity with the well-studied dipeptide (Dpp) permease of *Escherichia coli*. The solute binding protein (SBP) that mediates glutathione transport in *H. influenzae *is a lipoprotein termed GbpA and is 54% identical to *E. coli *DppA, a well-studied member of family 5 SBP's. The discovery linking GbpA to glutathione import came rather unexpectedly as this import-priming SBP was previously annotated as a heme-binding protein (HbpA), and was thought to mediate heme acquisition. Nonetheless, although many SBP's have been implicated in more than one function, a prominent physiological role for GbpA and its partner permease in heme acquisition appears to be very unlikely. Here, we sought to characterize five representative GbpA homologs in an effort to delineate the novel GbpA-family of glutathione-specific family 5 SBPs and to further clarify their functional role in terms of ligand preferences.

**Results:**

Lipoprotein and non-lipoprotein GbpA homologs were expressed in soluble form and substrate specificity was evaluated via a number of ligand binding assays. A physiologically insignificant affinity for hemin was observed for all five GbpA homologous test proteins. Three out of five test proteins were found to bind glutathione and some of its physiologically relevant derivatives with low- or submicromolar affinity. None of the tested SBP family 5 allocrites interacted with the remaining two GbpA test proteins. Structure-based sequence alignments and phylogenetic analysis show that the two binding-inert GbpA homologs clearly form a separate phylogenetic cluster. To elucidate a structure-function rationale for this phylogenetic differentiation, we determined the crystal structure of one of the GbpA family outliers from *H. parasuis*. Comparisons thereof with the previously determined structure of GbpA in complex with oxidized glutathione reveals the structural basis for the lack of allocrite binding capacity, thereby explaining the outlier behavior.

**Conclusions:**

Taken together, our studies provide for the first time a collective functional look on a novel, *Pasteurellaceae*-specific, SBP subfamily of glutathione binding proteins, which we now term GbpA proteins. Our studies strongly implicate GbpA family SBPs in the priming step of ABC-transporter-mediated translocation of useful forms of glutathione across the inner membrane, and rule out a general role for GbpA proteins in heme acquisition.

## Background

ATP-binding cassette (ABC)-transporters exist in all three kingdoms of life and transport a large variety of substrates across biological membranes. In addition to their well-documented role in solute transport, a diversity of sensory functions have been assigned that implicate ABC-transporters in the maintenance of cell integrity, responses to environmental stresses, cell-to-cell communication and cell differentiation and in pathogenicity. Based on the direction of transport, ABC transporters can be classified as either exporters or importers. Both classes are characterized by the coupling of two nucleotide-binding domains (NBD) and two transmembrane domains (TMD). In the case of ABC importers, which are found exclusively in prokaryotes, a fifth domain, termed the solute binding protein (SBP), is part of the functional unit [1]. SBPs bind their ligands with high affinity and deliver them to the permease unit (the TMDs), where the substrate is released into the translocation pore upon ATP binding and hydrolysis in the NBDs [2,3]. SBPs are located in the periplasm of Gram-negative bacteria, or lipid-anchored to the cell wall, or fused to the TMD in the case of Gram-positive bacteria and Archaea [4]. Although SBPs of Gram-negative bacteria exist predominantly as stand-alone periplasmic proteins, they are sometimes connected in a fusion protein with the TMD [4] or observed lipid-anchored to the inner membrane [5,6]. The physiological relevance of the immobilized versions of SBPs remains largely unaddressed in the literature.

Based on sequence homology analyses, the bacterial SBP superfamily has been classified into 8 clusters, with cluster 5 comprising dipeptide binders (DppA family), oligopeptide binders (OppA family) and nickel specific SBP's (NikA family) [7]. Continuous family updates by the Transporter Classification Database http://www.tcdb.org has now led to a cluster 5 SBPs containing up to 27 different subfamilies that are associated with translocation cargos as diverse as - in addition to di-and oligo-α-peptides and nickel substrates - antimicrobial peptides, δ-aminolevulinic acid, heme, plant opines, carbohydrates, the osmoprotective proline betaine, and the metal-chelater ethylene diamine tetraacetate. The most recent addition to the SBP family is termed GbpA (TCID: 3.A.1.5.27), a lipoprotein from *Haemophilus influenzae*, which binds reduced (GSH) and oxidized (GSSG) forms of glutathione to prime the dipeptide-DppBCDF ABC-transporter for glutathione translocation across the inner membrane [8]. Structural studies of the highly homologous GbpA from *H. parasuis *in complex with GSSG have revealed structural features that typify cluster 5 SBPs, namely, a pear-shaped, two-domain α/β-fold that collapses about the hinge region connecting the N-and C-terminal domains to sandwich the molecular cargo, in this case a single GSSG molecule [8]. In the absence of ligand, SBP's are flexible with the two domains rotating around the hinge and existing largely in the open conformation with both domains separated. Substrate binding induces the closed conformation, and the ligand is trapped at the interface between the two domains, according to what has been termed the "Venus Fly-trap" mechanism [9]. The structural analysis of GbpA in complex with GSSG has identified many specific interactions between GSSG and its cognate SBP that may be helpful in the delineation of the entire GbpA family [8]. The discovery that GbpA mediates glutathione transport in *H. influenza *came as a complete surprise as this protein was previously thought to be a heme-binding protein, accordingly annotated HbpA, and was implicated as a binding-platform for heme [5,10]. Nonetheless, GbpA does bind hemin, albeit weakly with an apparent *K*_d _of 655 μM [8], and a possible role for GbpA and DppBCDF in heme acquisition has been described [10,11]. In this regard, GbpA presents itself as a good example of the high degree of substrate promiscuity especially common among cluster 5 SBPs [12-15].

In light of our recent report on the functional reannotation of HbpA to GbpA [8], the present study was designed to elucidate further and refine this emerging SBP subfamily of glutathione-binding proteins and to clarify the roles of such proteins in glutathione and heme acquisition. GbpA homologs were identified employing BLAST and their clustering in the novel GbpA family was established based on structure-based motif fingerprinting. To ascertain the GbpA family functionally, we subsequently explored the ligand preferences of five representative GbpA homologous proteins. As the GbpA from *H. influenzae *is lipidated *in vivo*, we also incorporated in our test protein set GbpA homologous sequences that were not preceded by a peptidase II modifiable leader peptide, thereby providing the opportunity to uncover lipidation-dependent functional effects. Our studies indicate that GbpA family members are exclusively found in the Gram-negative *Pasteurellaceae*, where they have evolved by gene duplication from a canonical DppA sequence to prime the transport of physiologically useful forms of glutathione. Our data on the other hand do not support a general role for GbpA family proteins in heme acquisition. Finally, a phylogentically distinct cluster of GbpA homologues was identified, which appears to lack binding capacity not only for glutathione and other peptide ligands, but heme as well, thus casting a new twist in the possible substrate preferences of GbpA-like proteins.

## Results and Discussion

**Figure 1:**
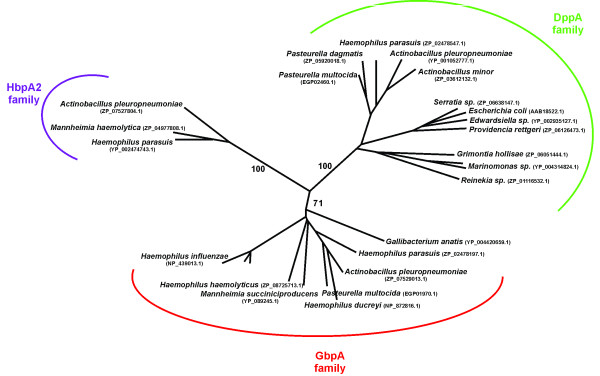
**Phylogenetic analyses of the top 100 GbpA homologs found in the National Center for Biotechnology Information (NCBI) microbial protein database reveal three distinct branches, clustering GbpA SBP's, canonical DppA proteins, and HbpA2 SBP's**. The phylogenetic tree was generated using the neighbor-joining tree construction method with Jukes-Cantor distances within the Geneious 5.3.4. software program and no outgroup was selected. Bootstrap resampling was conducted with 100 replicates by PhyML 3.0 [30] and support values for the three main nodes are provided. Representative sequences are shown by their NCBI Reference Sequence identifier.

**Figure 2:**
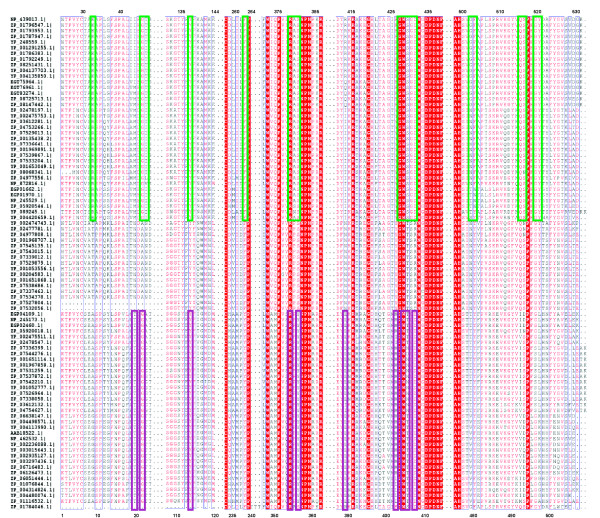
**Cut-and-spliced version of a hierarchical clustering-based multiple sequence alignment of the top-100 GbpA homologs found in the NCBI microbial protein database reveal invariant signature sequences for the GbpA and DppA subfamily**. Green-boxed residues of GbpA family members are ligand-interacting residues identified from the GSSG/GbpA_Hp _complex crystal structure (pdb id. 3M8U). Purple-boxed residues of DppA family members interact with the dipeptide allocrite in the *E. coli *DppA/glycylleucine complex structure (pdb id. 1DPP). Sequence names are NCBI Reference Sequence identifiers. The numbering on the top corresponds to the GbpA_Hp _sequence, while the numbering at the bottom refers to the DppA sequence of *E. coli*.

In order to delineate the GbpA family of SBP proteins and to identify GbpA homologs with signal peptidase II modifiable leader peptides, we BLASTed the GbpA_Hi _sequence against all available microbial databases in June 2011 http://www.ncbi.nlm.nih.gov/sutils/genom_table.cgi. We found GbpA homologs in 13 different species, all of which belong to the *Pasteurellaceae*, and more than half of these sequences (belonging to 7 species) were predicted lipoproteins by the LipoP 1.0 server http://www.cbs.dtu.dk/services/LipoP/. A survey of the top 100 homologs furthermore uncovered a number of established and predicted DppA proteins as well as several *Pasteurellaceae*-unique sequences that on first sight neither belong to the DppA-family, nor to the GbpA-family and that are all annotated as heme-binding proteins (HbpA). We will refer to these proteins with the affiliation HbpA2. These HbpA2 sequences were found in 3 species, *H. parasuis*, *M. haemolyticus*, and *A. pleuropneumoniae*, all of which also contained a GbpA. The identified GbpA, DppA, and HbpA2 proteins share at least 50% sequence identity, with the GbpA/HbpA2 couples being the closest relatives (all exceeding 60% sequence identity) and the DppA/HbpA2 couples having the most distant relationship. A phylogentic analysis using the Geneious 5.3.4 package http://www.geneious.com led to a striking delineation of these homologs into three clades, as supported by bootstrap resampling (Figure 1). Interestingly, most DppA proteins homologous to GbpA_Hi _are also found within the *Pasteurellaceae*, strongly suggesting that glutathione-specific GbpA proteins evolved paralogously in the *Pasteurellaceae *lineage from their canonical DppA dipeptide-binder. High-resolution crystal structures of liganded GbpA (in complex with GSSG) and DppA (in complex with glycylleucine) representatives have uncovered key ligand contact residues that provide family-specific signature sequences [8,16]. We highlighted such sequence fingerprints in a cut-and-spliced version of a hierarchical clustering-based multiple sequence alignment http://multalin.toulouse.inra.fr/multalin/ of the GbpAs' BLAST top 100 homologs shown in Figure 2. This analysis correlates strongly with the three clade separation, and reveals a strict conservation of 13 out of 18, and 8 out of 10 of the active site residues in the demarcated GbpA and DppA clade, respectively. Furthermore, Figure 2 highlights the versatility of the dpp-fold whereby a handful of key mutations on either side of the binding interface has led to a ligand-preference switch from a dipeptide to a disulfide bridge containing hexapeptide. Accordingly, the signature sequence for the GbpA family is more extended and comprises more residues than that of the DppA family.

Interestingly, the HbpA2 clade diverged significantly from both the GbpA and DppA signature sequences. In fact, some of the strictly conserved residues that contact the ligand's charged N- and C-termini in either the GbpA or the DppA family are replaced by physicochemically dissimilar residues in the HbpA2 sequences thereby virtually disrupting critical ligand-stabilizing salt bridges (In case of GbpA-GSSG binding, Arg33 substituted by a Thr, and Asp432 substituted by an Arg; in case of DppA-dipeptide binding, Asp408 substituted by an Arg). The ligand specificity of the HbpA2 clade is therefore difficult to predict, but it is highly unlikely that glutathione or dipeptides are the natural molecular cargos. Given the auxotrophic nature of *Pasteurellaceae *for heme and the fact that the dpp-architecture is a proven hemin-binding scaffold (*E. coli *DppA binds hemin with a 10 μM affinity [14] and also GbpA_Hi _displays an, albeit low, affinity for hemin [8]) it is tempting to speculate that HbpA2 proteins may play a role in heme transport.

To document the heme-binding characteristics of the GbpA family, to verify the role of the posttranslational 1,2-diacylglycerol-modification of GbpA proteins in terms of glutathione and heme binding, and to establish the ligand-preferences of the HbpA2 family, we selected in addition to GbpA_Hi _yet another GbpA lipoprotein (from *A. pleuropneumoniae*, GbpA_Ap_), 2 non-lipoprotein GbpA's (GbpA_Hp _and GbpA_Pm _from *H. parasuis *and *P. multocida*, respectively), and 2 HbpA2 proteins (HbpA2_Hp _and HBPA2_Ap _from *H. parasuis *and *A. pleuropneumoniae*, respectively), for further study.

**Figure 3:**
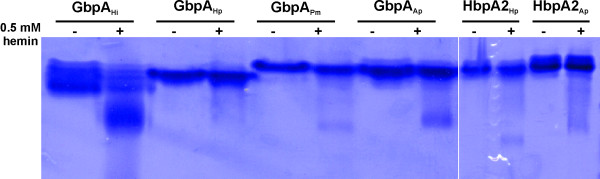
**Characterization of the hemin-binding properties of GbpA and HbpA2 family members**. Native-PAGE analysis of 10 μg test protein in the absence (-) or the presence (+) of 0.5 mM hemin. The faster migrating band for each test protein in the presence of hemin represented the hemin/protein complex as verified by heme-staining (see Materials).

In a previous report, we had employed a hemin-binding assay based on native-PAGE to show that GbpA_Hi _has a physiologically irrelevant affinity for hemin [8]. To probe heme-binding among our protein test panel, the purified recombinant soluble forms of the test proteins were subjected to our native-PAGE-based assay in the presence and absence of 0.5 mM hemin (Figure 3). As this hemin concentration approaches its *K*_d_-value, the GbpA_Hi _band splits up, with about half of it migrating faster because of complexation with hemin (as judged by visual inspection (red-brownish bands) and heme-staining with 2,3',5,5'-tetramethylbenzidine/H_2_O_2_). Although all tested proteins displayed the split migration pattern, the fraction of the faster running hemin-complexed bands is much lower compared to that of GbpA_Hi _and therefore indicative of an extremely low affinity for hemin. These results strongly suggest that heme-binding is not a general feature of the GbpA and HbpA2 family. Notably, the second best binder of hemin is GbpA_Ap_, the other lipoprotein in our test panel.

A fluorescence-based thermal shift assay (Thermofluor assay [17]) was subsequently employed to screen for potential allocrites. The set of putative ligands amounted to 15 different ligands comprising of glutathione, some of its derivatives, and already established allocrites of the type 5 SBP superfamily such as di- and tripeptides, δ-aminolevulinic acid, nickel, and proline-betaine. The temperature-induced changes in relative fluorescence of 100 μg of test protein as a function of candidate ligands at 1 mM were recorded and those ligands that significantly affected the transition midpoint temperature (*T*_m_) of the apo-form (threshold was set at 1.5°C) are shown in Table 1 as a function of the corresponding Δ*T*_m_-values (apparent *T*_m_-differences in °C for the ligand-protein complexes relative to the uncomplexed proteins). Our analysis revealed an affinity of all tested GbpA proteins for (ranked according to descending Δ*T*_m_): GSSG > GSH > S-methylglutathione ≅ glutathione-cysteine disulfide. In addition, GbpA_Hp _and GbpA_Pm _also showed a minor but significant *T*_m_-shift in the presence of the bulky S-alkylated glutathione derivatives, S-hexylglutathione and S-decylglutathione. Although certain S-modifications were tolerated, fragments of glutathione such as γ-glutamylcysteine or cysteinylglycine or a slightly elongated form of glutathione (homoglutathione) did not influence the melting behavior of any of the tested proteins, showing that the GbpA family carries a specificity for the glutathione backbone. Interestingly, in contrast to the notion that increasingly bulkier S-alkylations abrogate binding, the disulfide of glutathione with another glutathione molecule or with cysteine appear to be good allocrites for the entire GbpA family, strongly suggesting that the GbpA-fold evolved to bind these types of glutathione derivatives *in vivo*. This observation makes sense as many *Pasteurellaceae *are glutathione as well as cysteine auxotrophs and glutathione-cysteine disulfide reaches levels similar to those of glutathione in human plasma (up to 10 μM [18]).

**Table 1:** Summary of results obtained from thermal shift assays for the identification of GbpA- and HbpA2-family ligands out of a test set of typically family 5 SBP allocrites.

	**GbpA**_**Hi**_	**GbpA**_**Ap**_	**GbpA**_**Hp**_	**GbpA**_**Pm**_	**HbpA2**_**Hp**_	**HbpA2**_**Ap**_
	***T***_**m **_**(°C)**^**a**^					

GSSG	17.5	14.0	16.0	20.5	-	-
GSH	12.5	11.0	14.0	13.0	-	-
S-methylglutathione	10.5	7.5	11.5	11.5	-	-
S-nitrosoglutathione	7.0	6.5	9.5	12.0	-	-
glutathione cysteine disulfide	10.0	6.0	7.0	12.0	-	-
S-hexylglutathione	-	-	4.5	6.5	-	-
S-decylglutathione	-	-	2.5	3.0	-	-
homoglutathione γ-glutamylcysteine cysteinylglycine glycylleucine glycylglycylcysteine δ-aminolevulinic acid proline-betaine nickel}	-	-	-	-	-	-

**^a^**Interacting ligands are identified by means of the respective Δ*T*_m_-values, the apparent *T*_m_-differences in °C for the ligand-protein complexes relative to the uncomplexed proteins. A negative sign indicates ligands for which the Δ*T*_m _is smaller than 1.5°C.

**Figure 4:**
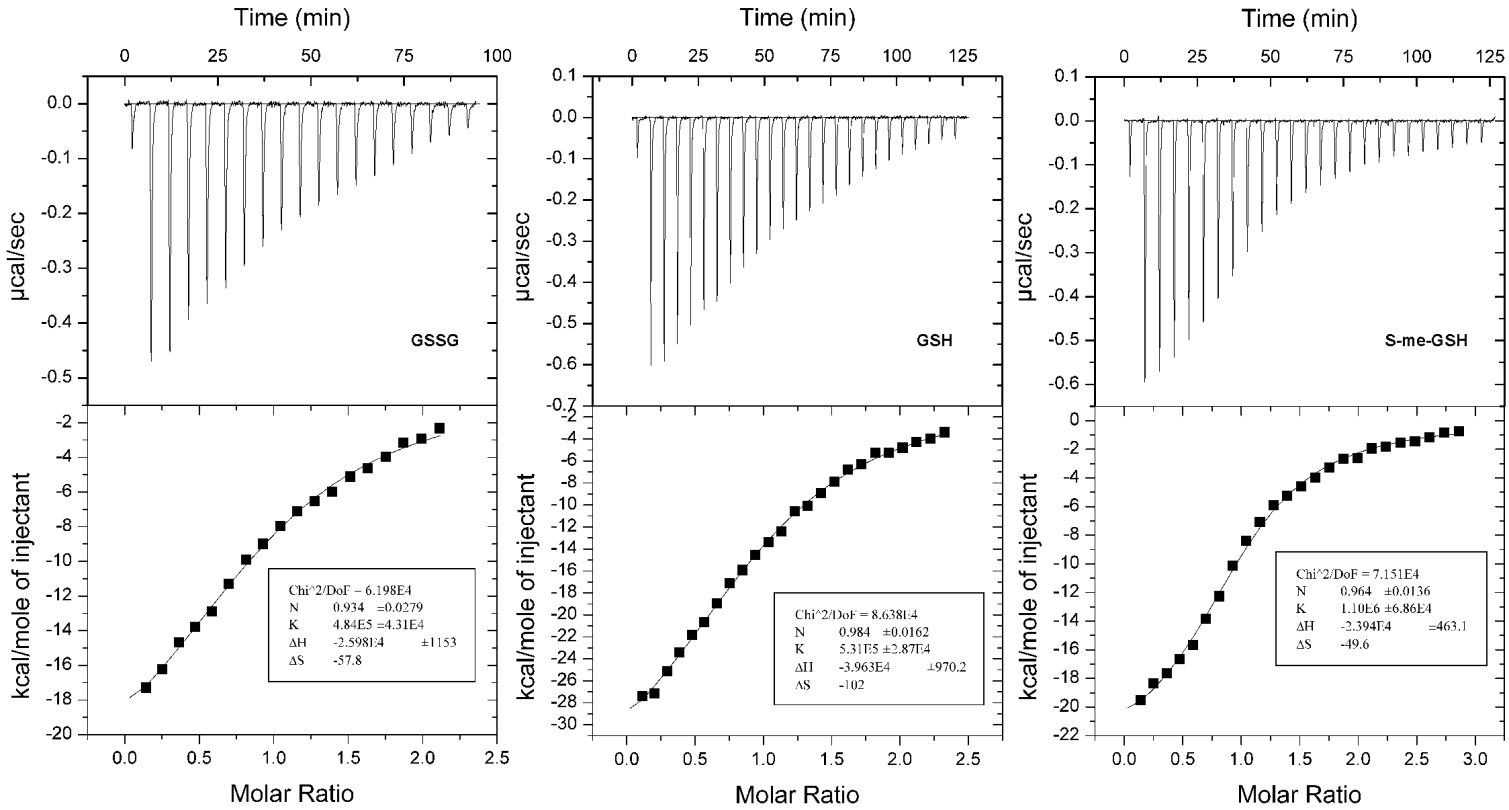
**Determination of the affinity constant of GbpA_Hp _for three different glutathione forms: the oxidized (GSSG), the reduced (GSH), and an S-derivatized form (S-me-GSH)**. Isothermal titration data for the titration of GbpA_Hp _with either GSSG, GSH, or S-me-GSH in 10 mMTris-HCl, pH 7.4. The upper panels show the calorimetric titrations for 10-μl injections with 300 s between injections. The lower panels represent the integrated heat values (from the upper panels) as a function of the protein/ligand molar ratio in the cell. The solid line represents the best fit of the single-site model to the experimental points.

Isothermal titration calorimetry (ITC) was subsequently used to determine the equilibrium dissociation constants for the interaction of our GbpA proteins with GSSG, GSH, and S-me-GSH. Typical ITC thermograms, showing the raw and integrated data for the interaction of GbpA_Hp _with these allocrites are shown in Figure 4, and all respective calculated *K*_d_-values are summarized in Table 2. Except for GbpA_Hp_, the ranking of binding strength according to the thermal shift Δ*T*_m_-values was recapitulated by the ITC-derived *K*_d_-values. Notably, affinities for the natural allocrites, GSSG and GSH, varied 200-fold, and for the artificial ligand S-me-GSH ~ 400-fold among the selected GbpA-family members, with GbpA_Hi _being the worst binder for all tested putative ligands. Interestingly, GbpA from *H. influenzae*, which naturally exists in a membrane anchored form, takes a unique position within the GbpA-family as the best binder of hemin, and the worst binder of glutathione. On the other hand, the soluble form of the predicted lipoprotein GbpA from *A. pleuropneumoniae *displays affinities for the tested glutathione derivatives that are similar to the two non-lipoprotein GbpA's (see Table 2). Therefore, membrane-anchoring of GbpA proteins appears not to impose any functional implications. Interestingly, the best hemin-binders from our test proteins were the lipoprotein GbpAs (Figure 3), amongst which the one of *H. influenzae *was shown to be biologically significant for heme acquisition [10,11]. Therefore, membrane-anchoring may influence the role of GbpAs in heme acquisition by increasing their intrinsic affinity for hemin. Nonetheless, GbpA-mediated heme import appears to be of minor importance under laboratory conditions as yet another family 5 SBP, the antimicrobial peptide binder SapA, has recently been shown to be essential for heme utilization by iron-starved nontypeable *H. influenzae *cells [19].

**Table 2:** Summary of the dissociation constants for the interaction of our GbpA-family test set with the physiologically relevant glutathione forms (GSSG and GSH), and the artificial S-methylglutathione (S-me-GSH) as determined by ITC at 37°C.

	**GbpA**_**Hi**_^**a**^	**GbpA**_**Ap**_	**GbpA**_**Hp**_	**GbpA**_**Pm**_
	***K*_d _(μM)**			

GSSG	12.9 ± 0.3	0.33 ± 0.05	2.1 ± 0.2	0.15 ± 0.03
GSH	56.4 ± 3.0	1.58 ± 0.2	1.9 ± 0.1	0.26 ± 0.05
S-methylglutathione	212 ± 17	1.17 ± 0.05	0.90 ± 0.07	0.55 ± 0.04

^a^Values were taken from ref. [8].

**Figure 5:**
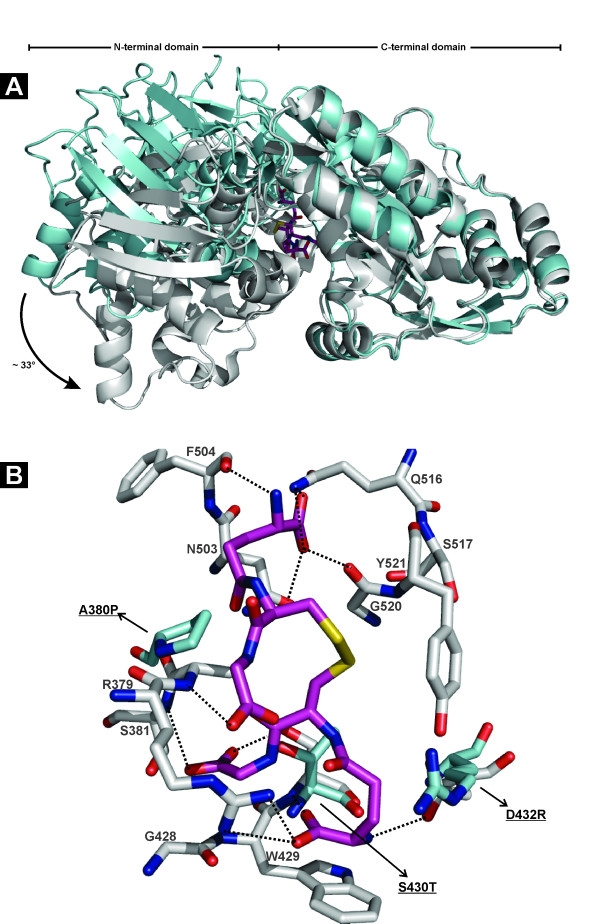
**Structure of HbpA2 from *H. parasuis***. (A) Ribbon diagram showing an overlay of GSSG-complexed GbpA (PDB id. 3M8U; gray) with HbpA2 from *H. parasuis *(PDB id. 3TPA; blue). The structures were superposed with respect to their C-terminal domains. HbpA2 shows a conformation that opens the cleft between the N- and C-terminal domains about 30° relative to its ligand-complexed paralogous counterpart. GSSG is depicted in atom-colored sticks. (B) Key binding residues of the GbpA C-terminal domain to accommodate GSSG (shown in atom-colored gray sticks) are replaced in HbpA2 by counterparts (shown in atom-colored blue sticks) that are incompatible with binding peptide-like allocrites. Residue numbering is according to PDB id. 3M8U. Some key interactions are depicted as black dashed lines. For clarity some interactions have been omitted. The figure was created with PyMOL (The PyMOL Molecular Graphics System, Schrödinger, LLC).

The lack of conservation of consensus sequence fingerprints important for ligand-binding by GbpA- and DppA-family proteins (Figure 2) had already suggested that the HbpA2 proteins in our test set would fail to bind the glutathione- and dipeptide-types of ligands. Indeed, our thermofluor analyses showed that none of the two HbpA2 proteins under study were able to interact with any of the tested type 5 SBP superfamily allocrites (Table 1). Because of the possibility that the HbpA2 proteins would co-purify with their natural ligands, as observed for some other structurally characterized SBP's, such as e. g. the cysteine-complexed CjaA from *Campylobacter jejuni *[20] or the oligopeptide-binder AppA from *Bacillus subtilis *in complex with a nonapeptide [21], we sought to determine the crystal structure of HbpA2_Hp _hoping to elucidate an interaction with a possible ligand. The crystal structure of HbpA2_Hp _was determined to 2.0 Å resolution by maximum-likelihood molecular replacement (Figure 5; additional file 1, Table S1). The structure reveals the two-lobe α/β-fold architecture and β-strand topology typical for SBP-like proteins, and is essentially identical to that of the structurally characterized GbpA and DppA proteins. Crystallographic refinement and exhaustive examination of residual difference electron density maps failed to provide any evidence for a bound ligand to HbpA2. Moreover, the N- and C-terminal domains were opened by about 33 degrees with respect to the GSSG-bound GbpA and glycylleucine-bound DppA reported previously [8,16] (Figure 5A), again indicating we crystallized apo-HbpA2_Hp_. Importantly, the crystal structure of HbpA2_Hp _offers an explanation for its inability to bind peptide-like ligands. Figure 5B shows a structural superposition of residues of the GbpA ligand-binding site with only those corresponding residues in HbpA2_Hp _of which the physicochemical properties are significantly different as revealed by our sequence alignments (Figure 2). This analysis focuses on the C-terminal lobe, because it comprised the majority of the ligand-interacting residues as shown by the GbpA_Hp_-GSSG complex [8] (13 out of 18 interactions), and due to the fact that it is believed to drive formation of the SBP-ligand-encounter complex [22]. Out of the 13 GSSG-contacting residues, 3 were not strictly conserved in HbpA2_Hp_, i.e. A380P, S430T, and D432R. All of these residues appear to be critical for GSSG-binding by GbpA_Hp_: the peptide-nitrogen of A380 hydrogen-bonds with the carbonyl oxygen of GlyI of one of the glutathione legs (GS-I); the D432 side chain carboxylate forms a salt bridge with the amino terminus of GS-I as well as H-bonds with the side chain hydroxyl groups of Y138 and Y521 thereby positioning these residues for favorable hydrophobic interactions, the side chain of S430 is involved in H-bonding with both the carboxylate- and amino-groups of the γ-glutamyl-moiety of GS-I [8]. The structural superposition in Figure 5B shows that S430 and D432 in the HbpA2_Hp _structure occupy the exact same position as the corresponding active site residues in GbpA_Hp_. At the same time, A380 takes a slightly different position which would be expected due to the elimination of the special structural role of a proline residue in maintaining loop structure at this position. Our structural analysis offers direct evidence that the A380P, S430T, and D432R substitutions would be grossly incompatible with GSH and GSSG binding as they would abolish electrostatic, H-bonding and hydrophobic interactions contributions critical for binding of such ligands. A similar analysis, this time against *E. coli *DppA, shows that R415 in HbpA2_Hp _takes the exact same position as the active site residue D408 in *E. coli *DppA, a residue that makes a salt-bridge with the amino-terminus of the bound dipeptide ligand. Thus, R415 would prevent dipeptide ligand binding. Finally, we note that the inability of the HbpA2 proteins to interact with either glutathione or dipeptides is correlated by looking at the interspecies occurrence: 2 out of 3 species with HbpA2 genes also carry genes for both a GbpA and a DppA family member.

## Conclusions

We here have provided a biochemical and phylogenetic delineation of the GbpA-family of glutathione-binding proteins. We showed that the GbpA proteins likely evolved exclusively within the *Pasteurellaceae *lineage by gene duplication from an already present dipeptide-binding protein, DppA, thereby explaining our previously reported functional annotation of GbpA proteins as periplasmic binding proteins that prime glutathione import to the cytoplasm via the cognate Dpp-ABC transporter [8]. GbpA proteins are specific for the glutathione backbone, but can tolerate S-modifications to different extends. This slightly promiscuous behavior probably resulted from the evolutionary tailoring of the GbpA scaffold to also accommodate useful disulfides of glutathione, such as GSSG and glutathione cysteine disulfide. Although GbpA proteins were formerly known as heme-binding proteins, an important implication of our work concerns the awareness that they clearly do not have a general role in heme acquisition. Apart from GbpA and/or DppA representatives, some *Pasteurellaceae *also carry the genetic information for a close, although phylogenetically distinct homolog, which we have termed HbpA2 in the present paper. Because we were unable to identify a molecular interaction partner for these paralogous HbpA2 proteins, their *in vivo *role will have to await further study. In any case, the current annotation as "heme-binding protein or HbpA" for HbpA2-family members is clearly inaccurate and databases should be rectified accordingly (e.g family 5 SBP with no known function).

## Methods

### Strains

Wild-type strain *H. influenzae *Rd was purchased from the American Type Culture Collection (Manassas, Va.). The *P. multocida *and *A. pleuropneumoniae *clinical isolates used in this study were a kind gift of Dr. Mario Vaneechoutte (Deptartment of Clinical Chemistry, Microbiology, and Immunology, University Hospital, Ghent, Belgium). The *H. parasuis *strain used in this study was isolated from the nasal cavity of a clinically healthy pig and was kindly provided by Dr. Filip Boyen (Department of Pathology, Bacteriology and Avian Diseases, Faculty of Veterinary Medicine, Ghent University, Belgium).

### Production and purification of recombinantSBP's

The construction of the expression plasmids pET-GbpAHi and pET-GbpAHp is described in ref. [8]. The remaining proteins in this study were overexpressed using similarly constructed plasmids also based on the pET20b vector template. The leader peptides of the respective proteins were predicted using the SignalP 3.0 server http://www.cbs.dtu.dk/services/SignalP/ and the LipoP 1.0 server http://www.cbs.dtu.dk/services/LipoP/ and replaced by the PelB leader peptide provided by the pET20b plasmid. In case of the *A. pleuropneumoniae gbpA *gene sequence, the codon that translates to the N-terminal Cys was furthermore replaced by the Met codon. Therefore the mature proteins started at positions 23, and 22 for the *P. multocida*, and *A. pleuropneumoniae *GbpA family members, respectively, and at positions 19, and 21 for the HbpA2 SBP's of *H. parasuis*, and *A. pleuropneumoniae*, respectively. All test protein coding sequences were extended with a his-tag to facilitate purification. The respective genes were PCR-amplified using forward and reverse primers (5' to 3'), respectively - with the cloning (restriction) site underlined and identified between brackets: GbpA_Pm _(CCATGGATAATAAAACCTTTATTAACTGC [*Nco*I], GCGGCCGCATCCGCTAACTTAGTGC [*Not*I]); GbpA_Ap _(CCATGGATGATAAAAATGCGGACG [*Nco*I], GCGGCCGCGTCGGCTAATTTTGTACCG [*Not*I]); HbpA2_Hp _(GATATCTCGGCACCGACAAATACATTG [*Eco*RV], CTCGAGTTAAGGCTTCAGACTTACGCCAT [*Xho*I]); HbpA2_Ap _(CCATGGCAGCGCCGGCACATACTTTAG [*Nco*I], GCGGCCGCTTCCGTTAGACTCACATTATAG [*Not*I]).

The proteins were expressed in *E. coli *and purified using a three-step chromatographic protocol (IMAC, followed by anion-exchange, and size-exclusion chromatography) as described in ref. [8]. The concentration of purified proteins was determined by the Bio-Rad Protein Assay with bovine serum albumin as the standard.

### Native PAGE heme-binding gel shift assay

Hemin stock solutions were prepared by dissolving bovine hemin chloride (Sigma-Aldrich) in 100 mM NaOH prior to 10-fold dilution in double distilled water. These solutions were then neutralized to pH 7.5 using HCl and filtered through a Millex-GP 0.22 μm filter unit (Millipore). Stock concentrations were determined spectrophotometrically (*ε*_385 _= 58,400 M^-1 ^cm^-1^), and the solutions were used within a day after preparation. Purified test protein (10 μg) was incubated with 0.5 mM hemin or water alone for 45 min at room temperature and subjected to native PAGE as described previously [8]. Hemin-complexed bands were visualized in-gel by their intrinsic peroxidase activity using 2,3',5,5'-tetramethylbenzidine and H_2_O_2 _[23]. The hemin-complexed protein species migrated faster compared to the apo-forms as was already described for other hemin-binding proteins [8,19,24].

### Thermal denaturation assays

Thermofluor thermal shift assays were conducted in a C1000 thermal cycler equipped with a CFX96 optical reaction module (Bio-Rad). The microplate wells were loaded with 25-μL solutions, containing 100 μg test protein, 2 × Sypro orange (Molecular Probes), and 1 mM of the test chemicals in 10 mM Tris-HCl, pH 8.0. The plates were sealed with Microseal B film (Bio-Rad) and heated from 30°C to 90°C at a rate of 2°C per min. The unfolding reactions were followed by simultaneously monitoring the relative fluorescence (FRET settings) using the charge-coupled device camera. The inflection point of the fluorescence versus-temperature curves was identified by plotting the first derivative over the temperature, and the minima were referred to as the melting temperatures (*T*_m_).

### Isothermal titration calorimetry (ITC)

Experiments were carried out using a VP-ITC MicroCalorimeter (MicroCal) at 37°C, and data were analyzed using the Origen ITC analysis software package supplied by MicroCal. Purified test proteins were dialyzed overnight against 10 mM Tris-HCl, pH 7.4, at 4°C. The resultant dialysis buffer was then used to dissolve the test compounds. Protein concentrations were measured spectrophotometrically using the respective theoretical extinction coefficients at 280 nm as calculated from the mature protein sequences at http://web.expasy.org/protparam/. GSSG concentrations were determined by the absorbance change at 340 nm resulting from the glutathione reductase-catalyzed NADPH-dependent conversion of GSSG to 2GSH (*ε*_340 _= 6,200 M^-1 ^cm^-1^). GSH concentrations were determined by the reaction with Ellman's reagent (*ε*_412 _= 14,000 M^-1 ^cm^-1^). All solutions were degassed prior to use. Titrations were always preceded by an initial injection of 3 μL and were carried out using 10-μL injections applied 300 s apart. The sample was stirred at a speed of 400 rpm throughout. Test compounds were always injected into the HbpA-containing sample cell. The heats of dilution were negligibly small for the titration of each ligand into buffer; hence the raw data needed no correction. The thermal titration data were fit to the one binding site model to determine the dissociation constant, *K*_d_. Several titrations were performed to evaluate reproducibility.

### Crystallization and structure determination of HbpA2 from *H. Parasuis*

Crystallization of HbpA2_Hp _(10 mg/mL in 10 mMTris-HCl pH 8.0, 100 mMNaCl) was screened using a Mosquito crystallization robot (TTP LabTech) based on 200 nL crystallization droplets (100-nL protein sample and 100-nL crystallization condition) equilibrated in sitting-drop geometry over 25-μL reservoirs containing a given crystallization condition. This led to the development of already well-formed rod-shaped crystals in condition 39 of the Hampton Research Index screen (0.1 M HEPES pH 7.0,30% v/v jeffamine ED-2001). This condition was optimized using a bigger "sitting-drop" geometry as follows. Crystallization droplets consisting of 1-μL protein sample and 1 μL 0.1 M HEPES pH 7.0, 30% v/v jeffamine ED-2001, were equilibrated against 0.75-mL reservoir solution containing 5-20% wt/v saturated ammonium sulfate. Diffraction quality crystals of HbpA2_Hp _grew overnight as clusters of easy separable crystalline rods (measuring 0.05 × 0.05 × 0.2 mm). For data collection under cryogenic conditions (100 K), single crystals were flash cooled with the help of a nylon loop directly in liquid nitrogen after a very brief incubation (typically < 30 s) in cryoprotecting solution containing 0.1 M HEPES pH 7.0, 30% v/v jeffamine ED-2001, and 20% v/v glycerol. The structure of HbpA2 from *H. parasuis *was determined by maximum-likelihood molecular replacement as implemented in the program suite PHASER [25]. The search model was prepared from the structure of *H. parasuis *GbpA in complex with GSSG [8] using the program Chainsaw [26], based on structure-based sequence alignments. The final search model contained alanines at all nonconserved positions and was stripped from all solvent molecules and ligand. The best solution was obtained in a combined search strategy whereby we searched for the C-terminal domain first. Inspection of electron density maps calculated with Fourier coefficients 2*F*_o_-*F*_c, MR_, α_c, MR _confirmed the solution as evidenced by extra density for missing structural elements and side chains. Model (re)building was carried out via a combination of automated methods as implemented in the PHENIX suite [27] and manual adjustments using the program COOT [28]. Crystallographic refinement and structure validation was carried out using PHENIX and Buster [27,29].

## Authors' contributions

BV designed the study and was involved in all experimental aspects of the work. AD contributed to recombinant protein production. RVdM and SNS carried out crystallographic studies. BV and SNS supervised the work and wrote the manuscript. All authors have read and approved the final manuscript.

## Supplementary Material

Additional file 1 **Table S1**. X-ray data collection and refinement statistics for HbpA2 of *H. parasuis*. Click here for file
