# Supplementary material for: Delineation of the Pasteurellaceae-specific GbpA-family of glutathione-binding proteins
Source: BMC Biochem. 2011 Nov 16;12:59. doi: 10.1186/1471-2091-12-59 (PMC3295651; doi:10.1186/1471-2091-12-59)
Supplement: Additional file 1 — Table S1. X-ray data collection and refinement statistics for HbpA2 of H. parasuis. [file 1471-2091-12-59-S1.DOC]

**Additional file 1**

**Table S1. Data collection and refinement statistics**

*Data collection*

Beamline/Wavelength (Å) PXIII/SLS, 1.0

Space group P2_1_2_1_2_1_

Unit cell parameters *a, b, c* (Å) 54.51, 61.04, 151.05

Unit cell parameters *α, β, γ* (°) 90.00, 90.00, 90.00

Resolution (Å) 50-2.0

Total reflections 196449

Unique reflections 34819 (5488)**^a^**

Completeness (%) 99.7 (98.6)

Rmeas (%) 13.0 (67.4)

I/σ(I) 12.16 (2.62)

*Refinement*

Resolution (Å) 40.64-2.0

R/Rfree (%) 16.27/20.79

Number of protein atoms 4095

Number of solvent molecules 612

Number of metal atoms 2

B-factors ( Å^2^) 27.29

rmsd

Bond length (Å) 0.010

Bond angle (°) 1.05

Ramachandran plot

Most favored region (%) 99.0

Allowed region (%) 1.0

PDB entry 3TPA

**^a^** Values in parentheses correspond to the highest-resolution shell
